# Strong as a Hippo’s Heart: Biomechanical Hippo Signaling During Zebrafish Cardiac Development

**DOI:** 10.3389/fcell.2021.731101

**Published:** 2021-08-05

**Authors:** Dorothee Bornhorst, Salim Abdelilah-Seyfried

**Affiliations:** ^1^Stem Cell Program, Division of Hematology and Oncology, Boston Children’s Hospital, Boston, MA, United States; ^2^Department of Stem Cell and Regenerative Biology, Harvard University, Cambridge, MA, United States; ^3^Institute of Biochemistry and Biology, University of Potsdam, Potsdam, Germany; ^4^Institute of Molecular Biology, Hannover Medical School, Hanover, Germany

**Keywords:** Hippo signaling, Yap1/Wwtr1 (Taz), cardiac development, mechanobiology, endocardium, myocardium, zebrafish, intra-organ-communication

## Abstract

The heart is comprised of multiple tissues that contribute to its physiological functions. During development, the growth of myocardium and endocardium is coupled and morphogenetic processes within these separate tissue layers are integrated. Here, we discuss the roles of mechanosensitive Hippo signaling in growth and morphogenesis of the zebrafish heart. Hippo signaling is involved in defining numbers of cardiac progenitor cells derived from the secondary heart field, in restricting the growth of the epicardium, and in guiding trabeculation and outflow tract formation. Recent work also shows that myocardial chamber dimensions serve as a blueprint for Hippo signaling-dependent growth of the endocardium. Evidently, Hippo pathway components act at the crossroads of various signaling pathways involved in embryonic zebrafish heart development. Elucidating how biomechanical Hippo signaling guides heart morphogenesis has direct implications for our understanding of cardiac physiology and pathophysiology.

## Introduction

**FIGURE 1:**
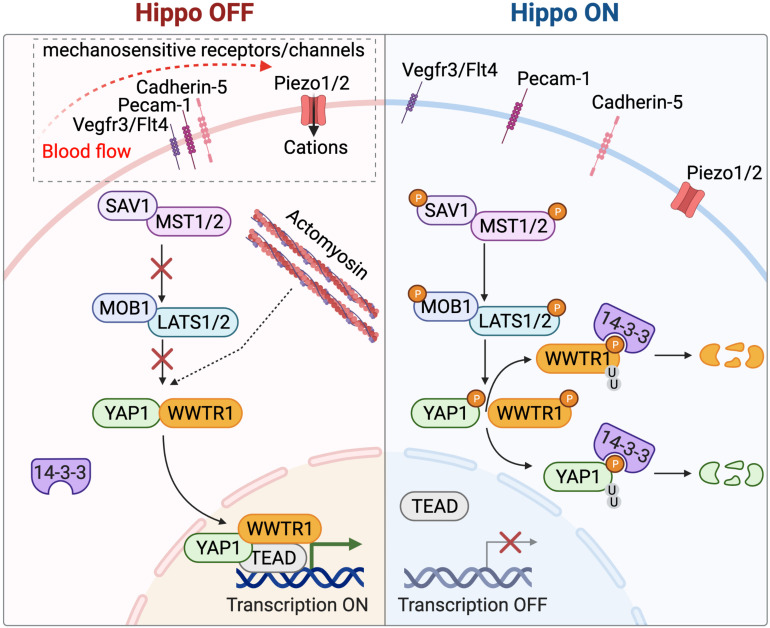
Biomechanical Hippo signaling pathways in the zebrafish endocardium. Biomechanical forces are sensed and transmitted by the mechanosensitive protein Cadherin-5 (Cdh5) and Piezo1/2 ion channels. This turns the Hippo signaling pathway to an inactive state (right) and results in the nuclear translocation of unphosphorylated YAP1/WWTR1 transcription factors where they form a complex with TEAD transcription factors to regulate gene expression. In the absence of biomechanical stimuli, the Hippo signaling pathway is active (left). This initiates a cascade of phosphorylation events on MST1/MST2 and LATS1/LATS2 kinases, which phosphorylate YAP1/WWTR1 proteins that are retained in the cytoplasm or become proteolytically degraded.

The Hippo signaling pathway is a key regulator of organ size and cell proliferation (Justice et al., 1995; Xu et al., 1995) in response to mechanical tension [(Dupont et al., 2011) and reviewed in Low et al. (2014)]. When Hippo signaling is active, the two Mammalian Sterile 20-like kinases (MST)1/2 and their phosphorylation targets Large tumor suppressor kinases (LATS)1/2 are in a phosphorylated state (Hergovich et al., 2006; Yin et al., 2013; Figure 1). YAP1 and WWTR1 are direct phosphorylation targets of LATS1/2, which causes their 14-3-3-mediated cytoplasmic retention and SCF-mediated degradation (Zhao et al., 2007, 2010; Liu et al., 2010). This prevents proliferative growth within the tissue or organ. Under mechanical stress or conditions of tissue crowding, Hippo signaling is off and the YAP1/WWTR1 transcriptional co-activators remain unphosphorylated and localize to the nucleus. There they bind members of the TEAD transcription factor family and activate proliferative target gene expression [reviewed in Zhao et al. (2008), Meng et al. (2016)].

In vertebrates, Hippo pathway components are key regulators of cardiovascular development [reviewed in Xiao et al. (2016); Ma et al. (2019)] and cardiac regeneration [reviewed in Mia and Singh (2019), Zheng et al. (2020)]. In mice, inactivation of Hippo pathway components in the developing heart caused cardiomyocyte overproliferation and increased size of the heart (Heallen et al., 2011). Consistently, overexpression of YAP within cardiomyocytes had the same effect (Von Gise et al., 2012). The transcriptional co-activators YAP1/WWTR1 (TAZ) are also involved in epicardial development and cell proliferation, epithelial-to-mesenchymal transition (EMT), and epicardial cell fate specification (Zhang et al., 2014; Singh et al., 2016). During murine cardiac cushion development, YAP1 potentiates TGF-driven Smad signaling, which regulates expression of the EMT-regulating target genes *Snail*, *Slug*, and *Twist1* (Zhang et al., 2014). Mechanical force directed at nuclei of mouse embryonic fibroblasts and epithelial cells was sufficient for the nuclear translocation of YAP1 protein (Elosegui-Artola et al., 2017). Similarly, mechanical stretching of an epithelial monolayer composed of MCF10A and MII cells *in vitro* induced entry of YAP1/WWTR1 into the nucleus and stimulated proliferation, which depended on the shape or the rigidity of the surrounding extracellular matrix (Aragona et al., 2013). This finding suggests that external strain forces can overcome the inhibition of YAP1/WWTR1 in growth-arrested or contact-inhibited cells. In zebrafish endothelial cells, Yap1 controls proliferation in response to blood flow and is essential for blood vessel maintenance (Nakajima et al., 2017). In this context, F-actin and Angiomotin affected the fluid flow-induced nuclear localization of Yap1. The apical polarity Crumbs complex with its associated proteins and cell junctional proteins can affect Hippo signaling [reviewed in Ma et al. (2019), Martin et al. (2021)]. The tight junctional protein Zona Occludens 2 (ZO-2) responds to high compression or tensile forces and directly interacts with YAP1/WWTR1 when these proteins shuttle between cell junctions, cytoplasm, and nucleus (Oka et al., 2010, 2012). Stretch-induced YAP1/WWTR1-dependent proliferation of murine endothelial cells was regulated by the mechanosensitive junctional protein Cadherin-5 (VE-Cadherin), indicating that mechanical stimulation at cell-cell junctions acts as a proliferative cue (Neto et al., 2018).

Zebrafish has evolved as a powerful model organism to study the role of mechanobiology during cardiovascular development. Zebrafish embryos can survive without a heartbeat throughout early development (Sehnert et al., 2002). This has facilitated experimental procedures with an altered cardiac contractility or blood flow that are not possible in other vertebrates. For instance, the formation of cardiac valves in response to blood flow [reviewed in Steed et al. (2016a), Paolini and Abdelilah-Seyfried (2018)], the delamination of myocardial cells during trabeculation (Staudt et al., 2014; Jiménez-Amilburu et al., 2016; Lai et al., 2018; Priya et al., 2020), or the development of proepicardium (Serluca, 2008; Peralta et al., 2013, 2020; Andrés-Delgado et al., 2019, 2020) have been subjects of studies with unprecedented cellular resolution.

**FIGURE 2:**
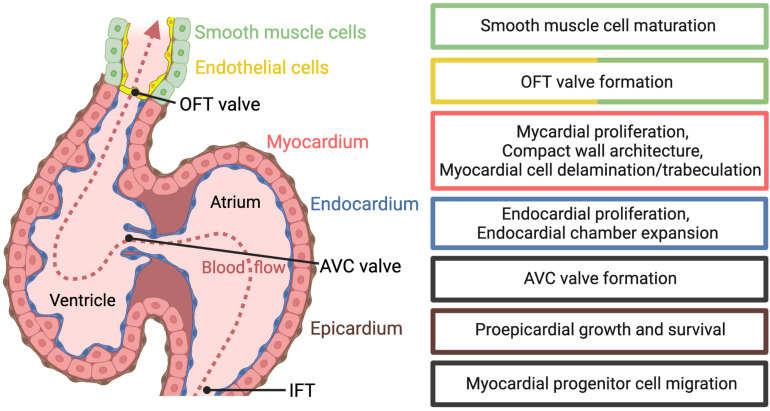
Hippo pathway-dependent developmental processes during early cardiac development in zebrafish. The zebrafish heart tube at 5 days post fertilization consists of the inner endocardium (blue), myocardium (red), and the surrounding epicardium (brown). The atrioventricular (AV) valve, comprised of myocardial and endocardial cells, and the outflow tract (OFT) valve, comprised of smooth muscle and endothelial cells, have formed and blood flow has a unidirectional flow pattern (dashed red line). The ventricular myocardium has segregated into inner trabecular and outer compact wall layers. Hippo pathways-dependent processes discussed in this review are highlighted in the colored boxes.

The role of mechanobiology in zebrafish cardiac morphogenesis and cell fate specification has been the focus of several recent reviews (Haack and Abdelilah-Seyfried, 2016; Paolini and Abdelilah-Seyfried, 2018; Duchemin et al., 2019b; Gunawan et al., 2021). Here, we only review the latest findings on biomechanical Hippo signaling during zebrafish cardiac development (Figure 2). From these studies, a complex picture emerges of Hippo signaling interactions with other signaling pathways in various cardiac developmental processes.

## Hippo Signaling Affects the Accretion of Secondary Heart Field-Derived Cardiac Progenitor Cells Into the Atrial Chamber

The formation of the zebrafish heart involves the migration of cardiac progenitor cells from the lateral plate mesoderm toward the embryonic midline. This process and the formation of the nascent zebrafish heart cone has been focus of previous reviews (Staudt and Stainier, 2012; Haack and Abdelilah-Seyfried, 2016). Of note, during heart cone assembly, Yap1 signaling is required for the survival of endodermal cells (Fukui et al., 2014), an essential tissue layer on which cardiac progenitor cells migrate. Mutants lacking endoderm (George et al., 1997; Schier et al., 1997; Zhang et al., 1998; Alexander et al., 1999; Reiter et al., 1999) or sphingosine 1-phosphate (S1P) signaling (Kupperman et al., 2000; Osborne et al., 2008) result in cardia bifida, a condition in which the two bilateral cardiac progenitor cell populations fail to reach the embryonic midline. In that study, the authors found that S1P signaling within the endodermal layer is required for Yap1 activity and the expression of the Yap1 target gene *connective tissue growth factor a*. In turn, this growth factor is essential for the survival of the endoderm (Fukui et al., 2014). In a related study, Miesfeld and Link (2014) identified an involvement of Yap1 in cardiac progenitor cell migration. They generated a transgenic Yap/Wwtr1-Tead activity reporter line, which indicated that Hippo signaling was active during cardiac progenitor cell migration. When overexpressing a dominant-negative transgene that prevented the interaction between Yap1/Wwtr1 and Tead, cardiac progenitor cell migration to the midline was impaired (Miesfeld and Link, 2014).

Another recent study by Fukui et al. (2018) expanded on their earlier discovery that Yap1 signaling indirectly affects cardiac progenitor cell migration. Here, they discovered a direct role of Hippo signaling within the anterior lateral plate mesoderm among a subset of secondary heart field-derived cardiac progenitor cells. Yap1 was required in *hand2-* and *islet1*-expressing myocardial cells that migrate to the venous pole of the heart. Mutants lacking Lats1/2 kinases had significantly increased numbers of *islet1*-positive atrial myocardial cells while a loss of Yap1/Wwtr1 had the opposite effect. That study also employed a transgenic Yap1/Wwtr1-Tead activity reporter line, which showed enhanced activity in *lats1/2* double mutants. This suggested these kinases restrict Yap1/Wwtr1-Tead activation during early myocardial determination. The mechanism of Yap1/Wwtr1 activity involved the upregulation of *hand2* and *bmp2* expression, which resulted in the phosphorylation of Smad1/5/9 in myocardial progenitor cells. Conversely, loss of Lats1/2 caused increased *hand2* expression and increased numbers of myocardial progenitor cells at the venous pole. Hence, these functional studies suggest, Hippo signaling restricts the accretion of myocardial progenitor cells to the venous pole by suppressing Yap1/Wwtr1-dependent Bmp2b signaling and *hand2* expression (Fukui et al., 2018). However, whether this process is dependent on mechanical stimuli within the anterior lateral plate mesoderm that regulates cell migration toward the venous pole of the heart will be an exciting topic for further investigations. Of note, Hand2 is a regulator of Fibronectin expression (Garavito-Aguilar et al., 2010), which is an important substrate for cardiac progenitor cell migration during early cardiogenesis (Trinh and Stainier, 2004). Loss of Hand2 causes cardiac bifida and increased *fibronectin* expression. Molecularly, Hand2 negatively regulates Fibronectin signaling to establishes a favorable milieu for cardiac fusion (Garavito-Aguilar et al., 2010). Hence, it is plausible that Hippo-Hand2-Fibronectin signaling may represent a second pathway controlling early cardiac progenitor cell migrations.

## Tensile Forces Trigger Yap1-Dependent Endocardial Cell Proliferation

During cardiac ballooning, chamber dimensions approximately double in size. Within the myocardial layer, this is due to the accretion of progenitor cells to both poles of the heart (inflow and outflow tract) (de Pater et al., 2009; Hami et al., 2011; Lazic and Scott, 2011; Zhou et al., 2011) and cell size increases (Auman et al., 2007; Samsa et al., 2015). However, endocardial chamber dimensions increase due to proliferation without a substantial accretion of external progenitor cells (Dietrich et al., 2015). Previous studies did not address how endocardial cell proliferation is triggered.

**FIGURE 3:**
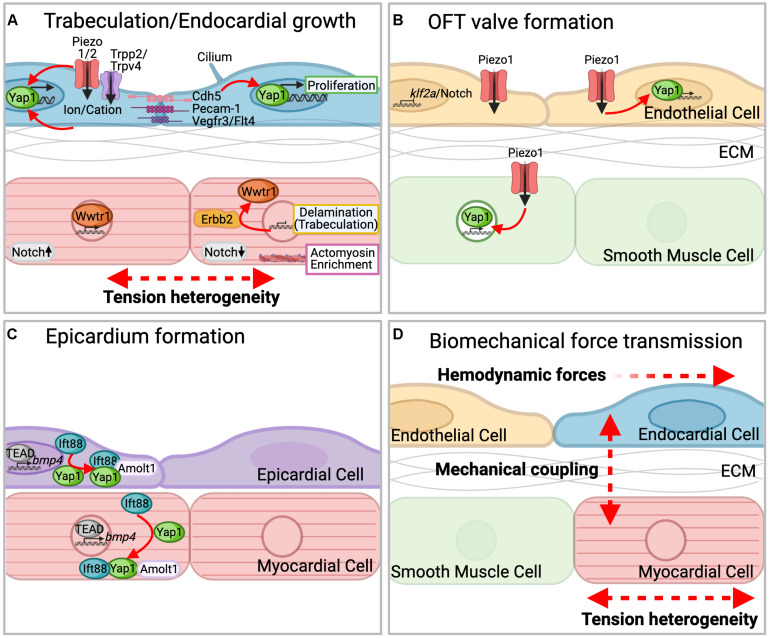
Hippo signaling pathways during zebrafish cardiac development. **(A)** Cell junctional complexes (including Cadherin-5, Pecam-1, and Vegfr3/Flt4), stretch-induced ion channels Piezo1/2, or primary cilia act as mechanosensors within the endocardium (blue). Increased junctional forces within the endocardium are sensed by Cadherin-5, which triggers the nuclear localization of Yap1/Wwtr1 and causes endocardial cell proliferation. A compact wall architecture is organized within the myocardium (red). Tension heterogeneity upon myocardial proliferation induces actomyosin enrichment at their apical sides and causes the nuclear localization of Yap1. This triggers delamination of myocardial cells that seed the trabeculation layer. Neighboring myocardial cells activate Notch signaling, which inhibits actomyosin network contractility. Erbb2-mediated nuclear export of Wwtr1 causes myocardial cell lamination into the trabecular layer. **(B)** Within the outflow tract (OFT) valve, Piezo1 regulates Yap1 nuclear localization within the endothelial (yellow) and smooth muscle cell layers (green). Within the endothelial layer, Piezo1 modulates expression of the mechanosensitive transcription factor *klf2a*. **(C)** During epicardial formation, Ift88 interacts with Yap1 within the cytoplasm and forms a complex with Amolt1 to regulate Yap1 activity. Within the nucleus a Yap1-Tead complex activates expression of *bmp4*, which restricts the growth of proepicardium (purple) and myocardium (red). **(D)** Biomechanical forces involve hemodynamics, biomechanical coupling, and tension heterogeneity acting within different tissue layers. These mechanical stimuli activate mechanosensitive Hippo pathway signaling.

A recent study addressed the role of Yap1 during endocardial chamber expansion. Using a laser-dissection approach (Behrndt et al., 2012), the authors first quantified whether an expansion of myocardial chamber dimensions causes an increase of tensile forces on endocardial cell junctions (Bornhorst et al., 2019). When using zebrafish models with increased cardiac chamber dimensions [overexpression of Wnt8a (Dohn and Waxman, 2012) or loss of Nkx2.5/2.7 (Targoff et al., 2008, 2013)], they detected higher junctional forces within the endocardium. This triggered nuclear localization of the Hippo pathway transcriptional regulators Yap1/Wwtr1 and increased endocardial cell proliferation. The authors also found that the force transmission within the endocardial tissue layer required the endothelium-specific adherens junction protein Cadherin-5 (VE-Cadherin) (Figure 3A). Upon loss of Cadherin-5, nuclear localization of Yap1 and endocardial cell proliferation was reduced in models with increased cardiac chamber dimensions (Bornhorst et al., 2019). Consistent with this finding, biomechanical forces due to blood flow also trigger the Yap1-dependent maturation and proliferation of hematopoietic stem and progenitor cells in zebrafish (Lundin et al., 2020).

While this work suggests some mode of intra-organ communication by which myocardial expansion is transmitted to the endocardium, the precise mechanism of that crosstalk has largely remained unexplored. Endocardium and myocardium are connected by extracellular matrix (cardiac jelly), which may propagate mechanical tension between these tissues [reviewed in Haack and Abdelilah-Seyfried (2016), Monte-Nieto et al. (2020), Derrick and Noël (2021)] and trigger increased junctional forces within the endocardium. This may explain why the expansion of myocardial chamber dimensions results in Hippo pathway-dependent proliferation within the endocardium. Another equally interesting question is, how Cadherin-5 transmits tensile forces from endocardial cell junctions toward the nucleus. The molecular cascade may include cytoskeletal components or mechanosensitive linker proteins, such as Vinculin, or a connected nuclear pore that allows Yap1 to enter the nucleus (Barry et al., 2015; Elosegui-Artola et al., 2017). Further experimental evidence will lead to additional insights into the molecular components involved in mediating tensile force-triggered proliferation within the zebrafish endocardium and the potential modes of mechanical coupling between myocardium and endocardium.

## The Mechanosensitive Piezo1 Channel and Yap1 Signaling Modulate Outflow Tract Valvulogenesis

Mechanical forces caused by blood flow play a major role in the morphogenesis of cardiac valves. A recent study by Duchemin et al. (2019a) described outflow tract (OFT) valve formation in zebrafish and characterized mechanosensitive pathways including signaling by the Hippo effector Yap1 during this process (Figure 3B). Characterization of OFT morphogenesis is of particular biomedical interest since bicuspid aortic valve disease is the most common congenital heart defect (Hoffman and Kaplan, 2002). Similar to the morphogenesis of atrioventricular valves, a comparatively well-characterized process [reviewed in Steed et al. (2016a), Paolini and Abdelilah-Seyfried (2018)], the formation of OFT valves is highly sensitive to blood flow. The authors discovered that mechanosensing by the mechanically gated and endothelially expressed transient potential (Trpp)2 and Trpv4 channels (Köttgen et al., 2008) and the Piezo-type mechanosensitive ion channel component (Piezo1) (Coste et al., 2010; Li et al., 2014; Volkers et al., 2014) caused a delayed morphogenesis of OFT valves. Trpp2 and Trpv4 play similar roles during the formation of zebrafish atrioventricular valves (Heckel et al., 2015). In the OFT region, blood flow affected the expression of the flow-sensitive Klf2a (Vermot et al., 2009; Heckel et al., 2015; Steed et al., 2016b) and Notch (Samsa et al., 2015; Fontana et al., 2020) signaling pathways, which were strongly active in regions of highest shear stress. Yap1 was expressed in both, endothelial and smooth muscle cells surrounding the OFT. In *piezo1* or *trpp2* mutants, Klf2a reporter expression became activated in anterior and posterior regions of the valve endothelium, suggesting these channels inhibit *klf2a* expression in that tissue. Piezo1 also impacted the nuclear localization of Yap1 within the neighboring vascular smooth muscle cell layer. Since *klf2a* mutants have normal Yap1 localization and expression, this effect may be attributed directly to Piezo1, which is expressed at low levels also within the smooth muscle cell layer. Hence, Piezo1 apparently has two roles during OFT valve formation by regulating *klf2a* expression within the endothelium, and Yap1 localization during smooth muscle cell maturation. This is yet another example for the complexity of Hippo pathway activity within the multi-tissue comprising heart. These findings add Piezo1 to the repertoire of mechanosensitive modulators of Hippo signaling during cardiac valve formation in zebrafish (Duchemin et al., 2019a). However, the precise molecular mechanisms of that crosstalk are currently unknown and remain an exciting topic for future investigations.

## Heterogeneities in Myocardial Cellular Tension and Hippo Signaling Form the Trabeculation Layer and Compact Wall of the Zebrafish Embryonic Heart

Myocardial cells undergo several morphological changes during zebrafish cardiac wall maturation of the ventricular chamber. These involve cardiac trabeculation, a process of myocardial cell delamination from the chamber wall and their extension into the ventricular lumen where they form a network of interconnected trabecular ridges (Sedmera et al., 2000; Moormann and Christoffels, 2003; Peshkovsky et al., 2011; Staudt et al., 2014). Lai et al. (2018) investigated the role of the Hippo effector and transcriptional co-activator Wwtr1 in myocardial cell delamination (Figure 3A). They found Wwtr1 localizes mainly to myocardial cell nuclei of the compact wall rather than trabecular layer and its loss causes defective trabeculation. In mosaic studies, *wwtr1* mutant myocardial cells more frequently populated the trabecular layer compared with wild-type cells. These results suggested Wwtr1 is required within compact layer myocardial cells. Further investigations revealed that a loss of Wwtr1 impacted the organization of cortical actin networks and cell-cell junctions. Blood flow and cardiac contractility serve as potential upstream regulators of Wwtr1 localization. Evidence for this hypothesis stems from the finding that a loss of blood flow due to the knock-down of Troponin T type 2a causes increased nuclear Wwtr1 localization. Hence, biomechanical cues may influence the Hippo signaling pathway component Wwtr1, which changes the organization of cortical actin networks or cell-cell junctions. Strikingly, signaling by Neuregulin/Erbb2, another important regulator of trabeculation, caused the nuclear export of Wwtr1 in myocardial cells. These findings suggested Neuregulin/Erbb2-mediated nuclear export of Wwtr1 may drive myocardial cells into the trabecular layer (Lai et al., 2018). How this signaling axis impacts myocardial cell contractility during trabeculation remained an unanswered question.

A recent study by Priya et al. (2020) directly addressed this question and identified heterogeneous levels of tension within the myocardial layer as a critical factor in trabeculation (Figure 3A). The authors found that proliferation and crowding among myocardial cells led to heterogeneous tissue tension throughout the compact layer of the myocardium. This triggered myocardial cells to delaminate and seed the trabecular layer. Delaminating myocardial cells displayed an enriched actomyosin network on their apical side and cortical tension was higher in these cells compared to the non-delamination myocardial cells. Using two experimental setups, they found an increased proliferation of myocardial cells (after treatment with a Vitamin D analog) caused increased myocardial cell density, sufficient to promote delamination, and decrease of myocardial cell proliferation (after treatment with Erbb2/MEK inhibitors) abrogated delamination. Similarly, when the actomyosin contractile machinery was impaired, myocardial cells remained in the compact layer while stimulation of myocardial cell contractility triggered myocardial cell delamination. Hence, differences in the tensile states of myocardial cells trigger the delamination process. Loss of Neuregulin/Erbb2 signaling abolished myocardial cell delamination, which was rescued by overexpressing constitutively active myosin regulatory light chain 9 (Myl9). The overexpression of constitutively active Myl9 also enabled myocardial cells to delaminate in the absence of blood flow and cardiac contractility. Hence, differences in cellular actomyosin contractility provide an essential trigger for myocardial cell delamination in the absence of either Neuregulin/Erbb2 signaling or blood flow. The study also addressed what limits myocardial cells in their ability to delaminate from the compact layer. Those myocardial cells adjacent to delaminating neighbors activated Notch signaling and inhibited actomyosin network contractility. This reduced the number of delaminating cells and allowed the compact layer to maintain its architecture (Priya et al., 2020). This study elucidated how biomechanical forces direct myocardial cell fate decisions within the trabecular layer and demonstrated the ability for robust self-organization of the myocardium based on a combination of intrinsic and extrinsic biomechanical cues. How Erbb2 signaling interacts with the actomyosin cytoskeleton and regulates Wwtr1 localization requires further investigation.

## Hippo Signaling Restricts Epicardial and Myocardial Growth by Controlling Proteins Involved in Ciliogenesis

The epicardial layer surrounding the heart has significant roles in cardiac development [(Moore et al., 1999; Wu et al., 2013) and reviewed in Limana et al. (2011)] and regeneration [(Kikuchi et al., 2011; González-Rosa et al., 2012) and reviewed in Limana et al. (2011)]. During zebrafish development, this tissue derives from the proepicardium, a group of cells that arises from two cell clusters that are close to the atrioventricular canal and inflow tract of the heart. After their release into the pericardial cavity, fluid flows caused by the heartbeat move these cell clusters within the pericardial cavity until they attach to and seed on the myocardial layer (Peralta et al., 2013). This process requires BMP and Notch activities (Andrés-Delgado et al., 2019, 2020). A recent study by Peralta et al. (2020) addressed the role of Hippo signaling in proepicardial growth. They discovered that several intraflagellar transport (IFT) complex B proteins, essential for cilia function and transport of cilia components, control epicardial cell numbers via modulating the Hippo signaling pathway (Figure 3C). The *ift88* and *elipsa/ift54* mutants had increased epicardial and atrial myocardial cell numbers. This process was independent of their cilia functions, because another well-characterized cilia mutant (*iguana*), lacking all primary cilia, had reduced epicardial cell numbers. The authors discovered that a loss of Ift88 causes increased proepicardial *bmp4* expression and Hippo signaling. In an elegant pharmacological suppression study, the authors found that blocking the interaction between Yap1 and Tead (using the drug Verteporfin) (Liu-Chittenden et al., 2012), suppressed the increases in BMP activity in *ift88* mutant embryos. These findings suggested that IFT proteins require Yap1 activity to modulate BMP signaling in the proepicardium and myocardium. This activity restricts proepicardial and secondary heart field-derived atrial myocardial growth. Other ciliary proteins of the Nephrocystin family regulate YAP1/WWTR1 (TAZ) activity in human fibroblast, rat, and mouse cell lines (Habbig et al., 2012; Frank et al., 2013; Grampa et al., 2016). Further, the Hippo kinases MST1/2-SAV1 promote ciliogenesis in 293T cells and depletion of Mst1/2-Sav1 resulted in ciliopathy phenotypes in zebrafish (Kim et al., 2014). Given that dysfunctional cilia cause cardiac defects [reviewed in Klena et al. (2017)], elucidating the connection between ciliary proteins and the biomechanical Hippo signaling pathway in cardiac development and regeneration is a promising field for future research.

## Discussion

Recent studies have significantly expanded our understanding of mechanosensitive Hippo signaling during zebrafish cardiac development. Currently, a complex picture has emerged of cell-intrinsic and -extrinsic cues that modulate this biomechanical signaling pathway. Likewise, our knowledge of the repertoire of developmental roles of the Hippo effectors Yap1/Wwtr1 and their crosstalk with other pathways is steadily increasing. Biomechanical forces that can impact Hippo signaling include hemodynamics, cell stretching, cellular crowding/tension, junctional forces, mechanical coupling, and actomyosin cytoskeletal rearrangements (Figure 3D). Upon mechanical stimulation, mechanosensation and force transmission toward Hippo signaling proteins involves the cell junctional protein Cadherin-5 (Bornhorst et al., 2019) and the cation ion channels Piezo1/2 (Duchemin et al., 2019a). This causes changes in gene expression profiles involving the Hippo signaling pathway. Precisely how biomechanical signaling activates the Hippo pathway in the context of zebrafish cardiac development is largely unknown.

Studies in the developing zebrafish heart revealed that Hippo signaling in one tissue layer can be affected by the development of other cardiac tissues. Such a form of intra-organ communication between myocardium and endocardium was observed when an expansion of myocardial atrial chamber dimensions triggered increased endocardial cell proliferation (Bornhorst et al., 2019). Increased cardiac chamber dimensions generated junctional tensile forces within the endocardium. This was sensed and transmitted into endocardial cells by Cadherin-5, driving nuclear localization of Yap1 and initiating endocardial cell proliferation (Bornhorst et al., 2019). Further, Flinn and colleagues found that Yap1 has a role in scar tissue formation, which is comprised of multiple cell types including fibroblasts, epicardial cells, and macrophages, during zebrafish cardiac regeneration. Yap1 regulates factors that mediate extracellular matrix deposition and macrophage activity (Flinn et al., 2019). However, they did not observe any effect on cardiomyocyte regeneration. We still lack a complete overview of molecular pathways involved in connecting the different tissue layers, cell types, and whether biomechanically active extracellular matrix components are part of this Hippo pathway-dependent intra-organ communication.

The AVC and OFT valves are composed of specialized endothelial/endocardial cell types, each with different cellular responses in the context of biomechanical Hippo signaling. Within OFT endothelial cells, Piezo1/2 channels control Notch and *klf2a* activities and affect Yap1 activation (Duchemin et al., 2019a). Whether this signaling pathway is also relevant for AVC endocardial cells needs to be resolved. The depletion of the polarity protein Lethal (2) giant larvae affected Yap1 activity in cardiomyocytes and reduced their cell numbers. This caused an enlargement of cardiomyocytes and severe cardiac deficits including atrioventricular valvulogenesis defects (Flinn et al., 2020). However, these valvular deficits may be an indirect consequence caused by altered blood flow patterns in these mutants. Also, potential differences in cell identities between OFT and AVC valve cells have largely remained unexplored. Systematically comparing these different valvular cell types and their biomechanical signaling will help to better understand how cardiac valve leaflets are being shaped in response to biomechanical forces. Likewise, it is unknown whether Yap1 is regulated by blood flow in zebrafish endocardial cells in a manner similar to the regulation of Yap1 in zebrafish vascular (Nakajima et al., 2017) or OFT endothelial cells (Duchemin et al., 2019a). Currently, we can only speculate that the diversity of endothelial and endocardial cell types also relates to their sensitivity to blood flow and that differences in the activation of biomechanical signaling pathways causes distinct cell fates and behaviors.

Multiple studies indicated blood flow as a key player during trabeculation and myocardial wall maturation (Chi et al., 2008; Peshkovsky et al., 2011; Staudt et al., 2014; Jiménez-Amilburu et al., 2016; Lai et al., 2018; Priya et al., 2020). In the absence of blood flow, myocardial wall maturation and trabeculation were disrupted and Wwtr1 nuclear localization increased within myocardial cells of the compact wall (Lai et al., 2018). It is not well understood by which mechanisms the myocardium is able to sense blood flow and what mode of intra-organ-communication takes place to transduce blood flow-dependent physical forces between endocardium and myocardium. In particular, the role of the endocardium in that mechanosensitive signal transduction process is unknown.

It is very likely that Hippo pathway signaling is also regulated by biomechanical stimuli during cardiac development of higher vertebrates. The pioneering studies in zebrafish have been facilitated by the ease with which blood flow and cardiac contractions can be modulated in that model organism. It will be an exciting but far more challenging approach to elucidate whether Hippo pathway-dependent cellular and molecular biomechanical processes play roles during the development or physiology of the mammalian four-chambered heart. For instance, recent evidence suggests that changes to cardiomyocyte cytoskeleton, cell junctions, and extracellular matrix composition impact YAP nuclear localization and heart regenerative capacity in mice (Aharonov et al., 2020). Overall, a more profound insight into mechanosensitive Hippo signaling pathways during the development of different cardiac tissues will be critical for understanding heart function under physiological and disease-related conditions.

## Author Contributions

DB designed the figures and wrote the first draft. DB and SA-S revised, wrote, and edited the final manuscript. Both authors contributed to the article and approved the submitted version.

## Conflict of Interest

The authors declare that the research was conducted in the absence of any commercial or financial relationships that could be construed as a potential conflict of interest.

## Publisher’s Note

All claims expressed in this article are solely those of the authors and do not necessarily represent those of their affiliated organizations, or those of the publisher, the editors and the reviewers. Any product that may be evaluated in this article, or claim that may be made by its manufacturer, is not guaranteed or endorsed by the publisher.
